# The relationship between dialysis adequacy and serum uric acid in dialysis patients; a cross-sectional multi-center study in Iranian hemodialysis centers

**DOI:** 10.15171/jrip.2017.28

**Published:** 2016-12-09

**Authors:** Eghlim Nemati, Arezoo khosravi, Behzad Einollahi, Mehdi Meshkati, Mehrdad Taghipour, Shahin Abbaszadeh

**Affiliations:** ^1^Nephrology and Urology Research Center, Baqiyatallah University of Medical Sciences, Tehran, Iran; ^2^Atherosclrosis Research Center, Baqiyatallah University of Medical Sciences, Tehran, Iran; ^3^Faculty of Medicine, Baqiyatallah University of Medical Sciences, Tehran, Iran

**Keywords:** Dialysis adequacy, Hemodialysis, Uric acid

## Abstract

**Introduction:** Uric acid is one of the most significant uremic toxins accumulating in chronic renal failure patients treated with standard dialysis. Its clearance has not any exact relation with urea and creatinine clearance.

**Objectives:** The aim of this study was to investigate the relationship between adequacy of dialysis and serum level of uric acid in dialysis patients of some dialysis centers in Iran.

**Patients and Methods:** In this study 1271 hemodialysis patients who have been treated for more than 3 months were evaluated. Their information and examinations from their files in all over the country were gathered and analyzed using SPSS versin18.0.

**Results:** In this study, a significant relationship between dialysis duration and serum level of uric acid was not detected, however, a significant relationship between patients Kt/V and uric acid (R=0.43, *P*=0.029) was seen. Patients who had higher adequacy of dialysis had a higher level of plasma uric acid.

**Conclusion:** For better controlling of plasma uric acid level of hemodialysis patients, increasing of the adequacy of dialysis or its duration is not effective. Other modalities of decreasing of serum uric acid like, changing diet or lifestyle or medical therapy may be necessary.

**Implication for health policy/practice/research/medical education:**

In this research 1271 hemodialysis patients who have been treated for more than three months were included and the association between adequacy of dialysis and serum level of uric acid in some dialysis centers in Iran evaluated. No significant relationship in dialysis duration with serum level of uric acid was seen. However, there was a significant association between patients’ Kt/V and uric acid was detected.

## Introduction

High mortality and morbidity in hemodialysis patients have remained as a problem. Insufficient hemodialysis is one of the most important causes of morbidity and mortality in hemodialysis patients (1,2). The insufficient dose of hemodialysis increases the duration of hospitalization and costs imposed on the patients. The efficiency of hemodialysis is a significant index which estimates sufficient doses of dialysis for the patients with end-stage renal disease (ESRD). An efficient dialysis can reduce the rates of complications and care costs (3). A famous study of NCDS (National Cooperative Dialysis Study) proved that as much as the efficiency of dialysis is higher, complications of uremia in body organs are reduced (4). The effectiveness of hemodialysis is calculated by different methods which one of the most common methods is the calculation of Kt/V. Kt/V includes clearance of urea and duration of dialysis and distribution volume of urea in the body. This method is used from past times in researchers as an indicator of dialysis efficiency and its association with the rate of mortality and complications (1,2,5).

The results of various studies show that determining the effectiveness of dialysis by the method of Kt/V is a very appropriate method to obtain the sufficient doses of dialysis for patients. The efficiency of dialysis is considered appropriate when Kt/V in hemodialysis patients is more than or equal to 1.2 (5). The uremic syndrome is caused by an accumulation of metabolites which is usually filtrated by glomeruli. Some of these metabolites act as toxins when they accumulate more than average concentrations and are known as uremic toxins. Uric acid, xanthine, and hypoxanthine are some of porin metabolites, which accumulate in the plasma of patients with ESRD. Porin metabolites constitute an important group of uremic toxins. These toxins such as uric acid and hypoxanthine may cause damage to the structure of proteins and vitamin D (1,2,6,7). Also, these toxins have an important role in immune deficiency of hemodialysis patients (7). Furthermore, these metabolites may cause an escalation of anorexia and weight loss in hemodialysis patients (8).

## Objectives

Regarding the significant role of uric acid especially in hemodialysis patients and the significance of accurate monitoring of uric acid in these patients and regarding this fact that uric acid clearance during hemodialysis has no exact relation with a clearance of urea and creatinine (8), we decided to evaluate the association between efficiency and duration of hemodialysis (the length of the time, they had been on dialysis) with a level of uric acid.

## Patients and Methods

This cross-sectional multi-center study recruited dialysis patients of 53 medical centers in Iran between February 2011 and March 2012. Registered data including, demographic and clinical data of hemodialysis patients who were admitted to these medical centers used for the study. Patients with the following criteria entered the study; ESRD, age over the 18 years, passing at least 3 months of the dialysis onset, 4 hours dialysis for each session and 3 times a week. Moreover, the details of the tests and dialysis adequacy extracted from the patients’ medical records. Adequacy of dialysis in patients is defined as Kt/V. Kt showed creatinine clearance and duration of dialysis, and V represents the volume of distribution of urea in the body. Dialysis adequacy is calculated in different ways. In this study Kt/V was precisely calculated based on the registered data as follow; Kt/V = in (1-URR) and URR (urea reduction ratio) = 1-(post BUN/pre BUN). The adequacy of dialysis in patients was evaluated with the above formula and using laboratory and clinical data which were mentioned in the patients documents.

Adequacy of patients’ dialysis is considered adequate when the Kt/V is greater than or equal to 1.2 in each dialysis session. To better analysis, patient divided into three groups based on the Kt/V as group one (less than one), group two (1 to 1.21) and group three (more than 1.21). The study patients divided into two groups for uric acid more and less than 6 mg/dL. Dialysis duration (the length of the time, they had been on dialysis) was categorized as less than or equal to 12 months, 12.1 to 60 months, 60.1 to 120 months and more than 120 months.

### Ethics issues

The research followed the tenets of the Declaration of Helsinki. The research was approved by the ethical committee of Baqiyatallah University of Medical Sciences (Ethical code# 762).

### Statistical analysis

All continuous variables were analyzed for normal distribution before further statistical analysis with Shapiro-Wilk test using STATA software version 11.2. Differences in categorical variables were analyzed using the chi-square test or the independent *t* test for continuous variables. Pearson’s correlation test was employed for the association of normally distributed variables. If the difference was significant, then post hoc test (Bonferroni) was conducted. A univariate and multiple linear regression models were also employed to examine the association between uric acid and variants of sex, age, Kt/V and hemoglobin levels. A *P* value less than 0.05 was considered statistically significant. The distributional properties of continuous data were expressed as a mean ± standard deviation (SD). Frequency and percentage presented the categorical data. All statistical analyses were performed with SPSS version 18.0 software.

## Results

### Patient characteristics

**Figure 1:**
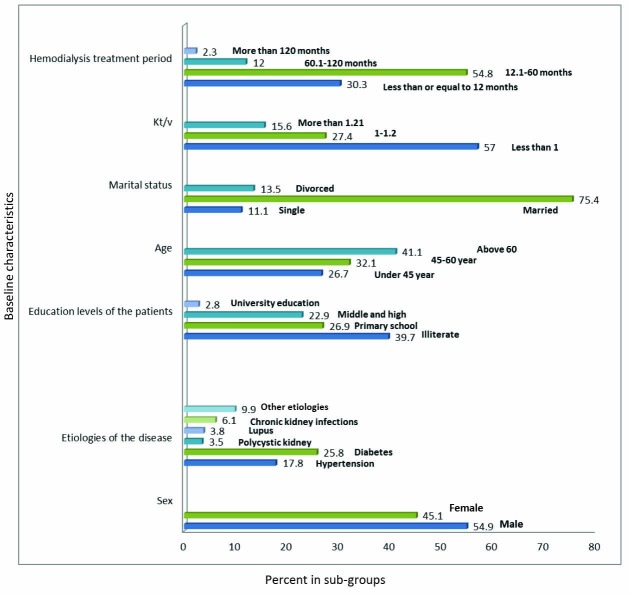
Demographic and clinical characteristics of participants at baseline.

A total of 1271 hemodialysis patients were evaluated in this study which 695 patients (54.9%) were male and 572 patients (45.1%) female. All of them were ESRD patients who were treated with hemodialysis. The primary cause of kidney disease was different in these patients and based on the gathered data, 213 patients (17.8%) did not know the cause of their disease. Etiology of other patients’ diseases is provided in Figure 1. The mean age of the patients was 54.56 ± 16.37 years. The average rate of URR was 58% ± 0.58. The average level of uric acid in the serum of the patients was 69.8 ± 1.86 mg/dL. The mean amount of Kt/V was 0.92 ± 0.315. A complete evaluation of laboratory variable and tests performed that the results gathered in Table 1.

**Table 1:** Laboratory and baseline characteristics of patients

**Laboratory tests**	**Mean ± SD**
Hb (g/dL)	10.30 ± 1.81
BUN-pre (mg/dL)	98.97 ± 53.99
BUN-post (mg/dL)	45.96 ± 33.03
Urea (mg/dL)	0.58 ± 0.14
Cr (mg/dL)	9.08 ± 3.46
Sodium (mEq/L)	139.13 ± 4.16
Potassium (mEq/L)	5.12 ± 0.78
Calcium (mg/dL)	9.13 ± 0.99
Phosphorus (mg/dL)	5.62 ± 1.66
PTH (ρg/mL)	451.46 ± 491.82
ALK-P (U/L)	388.33 ± 367.51
FBS (mg/dL)	133.23 ± 76.03
Kt/V	0.92 ± 0.31
Dialysis duration (months)	35.85 ± 35.61

### Relationships between gender and other clinical and laboratory characteristics

In this study, the mean of some clinical and para-clinic characteristics such as age, URR, Kt/V, uric acid levels and hemodialysis period was evaluated in both genders (male and female). Moreover, the statistical differences and correlations are presented in Table 2.

**Table 2:** Factors influencing dialysis adequacy according to gender

**Characteristics**	**Gender**		***P***
	**Female (mean±SD)**	**Male (mean±SD)**	
Age	‏54.33	55	<0.05
URR (%)	56 ± 0.144	60 ± 0.147	‏0.001
Kt/V	0.88 ± 0.303	0.97 ± 0.323	‏0.001
Serum uric acid level (mg/dL)	7.13 ± 1.89	6.78 ± 1.81	‏0.001
Hemodialysis duration (months)	36.7 ± 27.32	38.7 ± 29.5	<0.05
Serum uric acid level category (mg/dL)			
≤6	362 (27.5%)	628 (64.9)	‏0.001
>6	952 (72.5%)	361 (35.1)	‏0.001
Kt/V category			
‏<1	443 (61.4%)	278 (38.6%)	‏0.001
‏1-1.21	173 (49.7%)	175 (50.3%)	<0.05
>1.21	79 (39.9%)	119 (60.1%)	‏0.001

Regarding the URR rate — it was slightly more than males — it revealed that the dialysis adequacy of women in this study was little more than male.

### Correlation between factors effecting dialysis adequacy

In this study, we found a significant inverse correlation between age and serum uric acid level (*P* = 0.001, R = -0.86). Moreover, the study showed that older patients have relatively higher levels of uric acid that youngers.

Likewise, the correlation between acid uric and different age classes of the patients was as follows: in age group under 45 years old, 445 patients (72.8%) had uric acid levels more than 6 mg/dL and 166 patients (27.1%) serum uric acid with less than or equal to 6 mg/dL. In age group 45-60 years old, 540 patients (72.3%) with uric acid level more than 6 mg/dL and 207 patients (27.7%) had uric acid levels less than or equal to 6 mg/dL. In the case of age group above 60 years old, 535 patients (62.9%) had the uric acid level more than 6 mg/dL and the uric acid levels was less than or equal to 6 mg/dL in 316 patients (37.1%).

Accordingly, a significant inverse correlation of age and URR of the patients (*P* = 0.014, R = -0.70) was detected.

Additionally, the correlation between age and Kt/V of the patients was significantly inverse (*P* = 0.029, R = -0.62).

Thus, study showed that the efficiency of hemodialysis is lower when the patients are older. The correlation between the duration of hemodialysis (the length of the time, they had been on dialysis) with URR and Kt/V were not significant.

In this study patients were divided into four groups regarding the duration of their dialysis (the length of the time, they had been on dialysis). The association of each group with uric acid level of the serum was as follows:

Under 12 months: 460 patients (67.9%) with uric acid level more than 6 mg/dL and 217 patients (32.1%) less than or equal to 6 mg/dL. 12.1-60 months: 843 patients (70.1%) with uric acid level more than 6 mg/dL and 361 patients (29.9%) less than or equal to 6 mg/dL. More than 120 months: 50 patients (76.9%) with uric acid level more than 6 mg/dL and 15 patients (23.1%) less than or equal to 6 mg/dL. However, these differences were not significant (*P* = 0.09).

Accordingly, the correlation between URR and serum uric acid levels was not significant. However, a significant positive correlation between proportion of Kt/V and the level of serum acid uric (*P* = 0.029, R = 0.43) was detected. Hence, patients with higher dialysis efficiency had higher levels of serum uric acid.

In this study we also divided patients into three groups regarding the proportion of Kt/V. Table 3 shows the association of each group and levels of uric acid. The table shows that most of the patients with Kt/V above 1.21 had the uric acid level above 6 mg/dL (*P* = 0.41). Also, the total difference between groups was significant (*P* = 0.037). To sub-group analysis, the post hoc test was used, and the results are shown in Table 4.

**Table 3:** Serum uric acid levels and different categories of Kt/V ratio

	**Category**	**Mean±SD**	**Uric acid category**		***P***
			**UA ≤ 6 mg/dL**	**UA> 6 mg/dL**	
Kt/V	<1	6.73±1.86	260 (35.9)	465(64.1)	‏-
	1-1.2	7.00±1.86	108 (31)	240(69)	‏-
	>1.21	6.85±1.82	56 (28.4)	141(71.6)<1	‏0.41

**Table 4:** Post hoc test for evaluating the subgroups of Kt/V

	**(I) Kt/V** **category**	**(J) Kt/V category**	**SE**	***P***
Bonferroni	<1	1-1.2	0.118‏-	0.07
		>1.21	0.146‏-	0.23‏-
	1-1.2	<1	0.118‏-	0.07
		>1.21	0.162‏-	1.00
	>1.21	<1	0.146‏-	0.23‏-
		1-1.2	0.162‏-	1.00

Abbreviation: SE, standard error.

Furthermore, in male patients, a significant positive correlation of Kt/V and uric acid level was detected (R = 0.139, *P* = 0.001), however in female patients such association was not seen.

Finally in this study, linear regression test was conducted to assess the relationship between uric acid and variables of gender, age, Kt/V and plasma hemoglobin. Table 5 illustrates the relations remained significant after adjustment for variables of gender, age, Kt/V and plasma hemoglobin. However, in this method of analyzing, the association between age and uric acid levels of the patients was not significant.

**Table 5:** Linear Regression test adjustment for variables of gender, age, Kt/V and plasma hemoglobin

** Model**		** Coefficients **			**t **	**P**
		** Unstandardized Coefficients **		** Standardized coefficients **		
		** B **	** SE**	** Beta**		
1	Constant	6.409	0.432		14.83	0.001
	Gender	-0.234	0.111	-0.063	-2.102	0.036
	Age (years)	-0.006	0.004	-0.052	-1.744	0.081
	Kt/V	0.359 0.176	0.061	2.043	0.041
	Hb (g/dL)	0.085	0.031	0.082	2.768	0.006

## Discussion

Cardiovascular diseases and inefficiency of hemodialysis are the main factors for determining the morbidity and the mortality in the patients (2-6). The efficiency of hemodialysis is a major factor which estimates the sufficient dose of dialysis for the ESRD patients. Applying an effective dialysis can reduce the complications and the care costs (3). Renal physician association of the United States suggests periodic assessment of dialysis efficiency by dialysis clinics. NCDS proved that as much as the efficiency of dialysis is higher the complications of uremia in body organs is more reduced. The effectiveness of dialysis is measured by different methods which Kt/V is one of the most common methods. The results of different studies have shown that, determining the efficiency of dialysis by Kt/V method is a very appropriate way by which we can calculate the sufficient dose of dialysis for the patients. By applying an effective dialysis, patients can have a better life quality and the complications and the mortality of ESRD patients will be reduced. Additionally using Kt/V is preferred in comparison to URR since it reflects urea clearance more delicately (9,10).

Hence, the efficiency of dialysis is considered appropriate when the amount of Kt/V in each dialysis time is more than or equal to 1.2 (11). The uremic syndrome is a consequence of accumulating of metabolites which are commonly filtrated by glomeruli. Some of these metabolites act as toxins when they accumulate in the plasma more than the standard level and are known as uremic toxins. Detecting these toxins and determining their role will help to control the disease and to improve the patient’s health level. In fact, the main aim of dialysis is to reduce the serum levels of these toxins and to bring it to the normal levels. Also, these metabolites worsen anorexia and weight loss in dialysis patients (1-4). In the study conducted by Mousavi Movahed et al, dialysis efficiency in hemodialysis patients was investigated. The results showed that dialysis efficiency is significantly higher in women than men. The younger patients also had better dialysis efficiency (12).

Accordingly, Mogharrab et al found no significant association between patients’ age and dialysis efficiency, however their study showed that females had better dialysis efficiency (13). Present study showed no significant association between dialysis duration and dialysis efficiency indexes (Kt/V and URR). The association between duration of dialysis and uric acid level in serum was not significant.

Hakim et al studied the clearance of creatinine, urea and uric acid in the dialysis patients and showed, serum uric acid clearance during dialysis has no accurate association with the clearance of creatinine and urea (2). Silverstein et al in a study on 63 dialysis children measured their levels of serum uric acid. They also found no particular association between Kt/V and uric acid level (14). However, we found a significant direct association between Kt/V and uric acid level which implies, patients with higher Kt/V had a higher level of uric acid.

Additionally, Lesan Pezeshki et al, and also the result of the study by Taziki and colleagues, showed the proportion of certain metabolites of porin such as hypoxanthine are increased after dialysis (15,16). Bullo et al compared serum level of some metabolites of porin (hypoxanthine, ADP, and AMP) between dialysis patients and control group and showed that level of ADP and AMP in dialysis patients is normal, but hypoxanthine is higher than the control group (17).

Anorexia is one of the symptoms of the uremic syndrome and is caused by an accumulation of uremic toxins in plasma. Based on the study of Shabazian, levels of uric acid in dialysis patients reduced after hemodialysis, but this level either before the dialysis or after that was above normal limits. Likewise, Shabazian measured serum uric acid level before and after dialysis. The results showed that serum uric acid falls after dialysis (18). It is apparent that porin catabolism and production of uric acid is not limited to dialysis session duration and uric acid metabolism measured immediately after dialysis is not a proper indicator of uric acid accumulation in the plasma. Furthermore it should be mentioned that in some studies catabolism of porins increases after dialysis (17,19).

## Conclusion

The results show that improving the efficiency of dialysis may improve the quality of life and reduce the complications and costs of patients but it cannot help to control uric acid level. It is better to apply other modalities such as medical therapy or special diets or changing lifestyle to control it.

## Limitations of the study

Given that serum uric acid levels immediately before and after hemodialysis were not available in registered data, it was not possible to measure the uric, acid clearance and compare with urea and creatinine clearance. It should be noted that, this issue was not as a goal of the study.

## Acknowledgments

The present study is a part of a thesis approved by Baqiyatallah University of Medical Sciences. The authors thank the assistance of all participants in this study and also nurses of all hospitals for kindly cooperation.

## Authors’ contribution

All authors engaged in the design of the research and acquisition of information. BE, EN and AK planned the final manuscript. All authors reviewed, revised, and accepted the final manuscript.

## Conflicts of interest

The authors declare no conflict of interest.

## Ethical considerations

Ethical matters such as (plagiarism, misconduct, data fabrication, falsification, and double publication or submission) have been thoroughly controlling by all authors.

## Funding/Support

This paper is extracted from the thesis of Mehdi Meshkati. This study was supported financially by the deputy of research of Baqiyatallah University of Medical Sciences (Grant# 762, 2014).
